# Supplementary material for: A lncRNA Dleu2-encoded peptide relieves autoimmunity by facilitating Smad3-mediated Treg induction
Source: EMBO Rep. 2024 Jan 30;25(3):18. doi: 10.1038/s44319-024-00070-4 (PMC10933344; doi:10.1038/s44319-024-00070-4)
Supplement: Supplementary file 1 — Appendix [file 44319_2024_70_MOESM1_ESM.pdf]

Appendix content:

Appendix Fig S1	page2
Appendix Fig S2	page3
Appendix Fig S3	page4
Appendix Fig S4	page5
Appendix Fig S5	page6
Appendix Fig S6	page7
Appendix Fig S7	page8
Appendix Fig S8	page9

```
Mouse  GGAGCTTTGCTGAAACTGCACAAAAAATCGAGCTGGGGGGTTCCCTGGTC  98
Human  GGAGCTTTGCTGAAACTGCACAAAAAATCGAGCCGGGGGGTTCCCTGGTC  50
       ********************************* ****************

Mouse  CCCGATGTTGGGGCGGAGAGCGCGGCGCCGAGGGAGGGGGCGGCGCAGAC  148
Human  CCCGGCGATGGGGCGGGGAGCGCTGCGCCGGGGGAGGGGGCGG-------   93
       ****  * ******** ****** ****** ************

Mouse  CGGCCTA--------------------------GGGGACACCTGGTCGAG  172
Human  -------GCGCGGCGGCGCGGGCCCCGCCGAGGGGGGACACCTGGCTGAG  136
                                        ************  ***

Mouse  -CGCAGCCGCCGCTCCGCTCGAGCCCTGCGCTCCAGTGCCCCCACTGGCT  221
Human  GCACAGCTGCCGCCGTGCCTTTCCGCGCGAGCCCAGAGCTCCGATCCCTG  186
        * **** *****   **     * *      **** ** ** *
```

Appendix Fig S1: mRNA transcripts and ORF of Dleu2-17aa and DLEU2-25aa.

Sequence alignment of Dleu2-17aa and DLEU2-25aa DNA sequences, identical nucleotides are indicated with a star mark. The ORFs are highlighted in yellow.

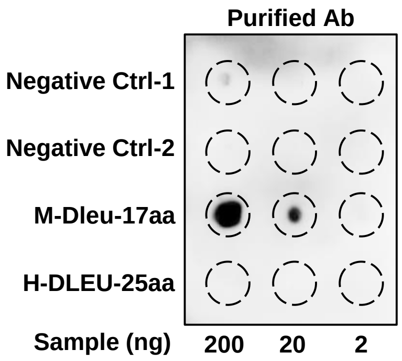

Appendix Fig S2: Verification of Dleu2-17aa antibody.

Immunoblotting of Dleu2-17aa and DLEU2-25aa using synthetic antibodies (n=3).

Data information: n indicates biological replicate.

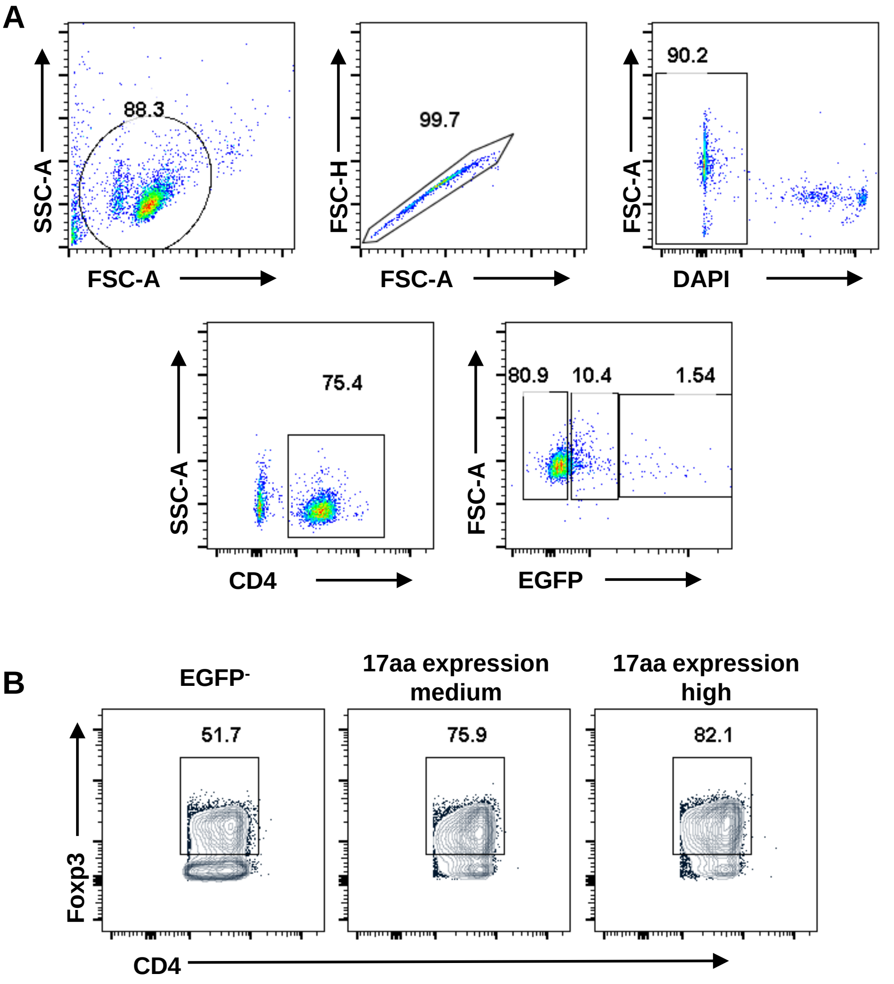

Appendix Fig S3: Dleu2-17aa overexpression induces iTreg differentiation

A. Gating strategy for sorting CD4^{+} T cells transfected with Dleu2-17aa overexpression vector based on enhanced green fluorescent protein (EGFP) intensity (n=3)..

B. Foxp3 staining in the sorted populations after 72 hours culture under iTreg conditions (n=3).

Data information: n indicates biological replicate.

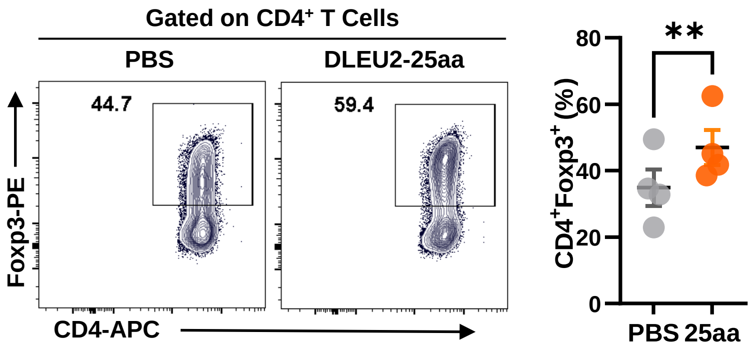

Appendix Fig S4: Effects of DLEU2-25aa on iTreg cell differentiation.

Phenotype and relative frequencies of Treg subsets in micropeptide treated naïve CD4+ T cells of WT mice. Sorted naive $CD4^+$ T cells were incubated with PBS or DLEU2-25aa at 10μM for 72 hours under iTreg induction conditions and Foxp3 expression in live cells were then analyzed (n = 4).

Data information: n indicates biological replicate. Error bars are mean ± SEM. **P<0.01. Statistical analysis was by two-tailed Student's t-test.

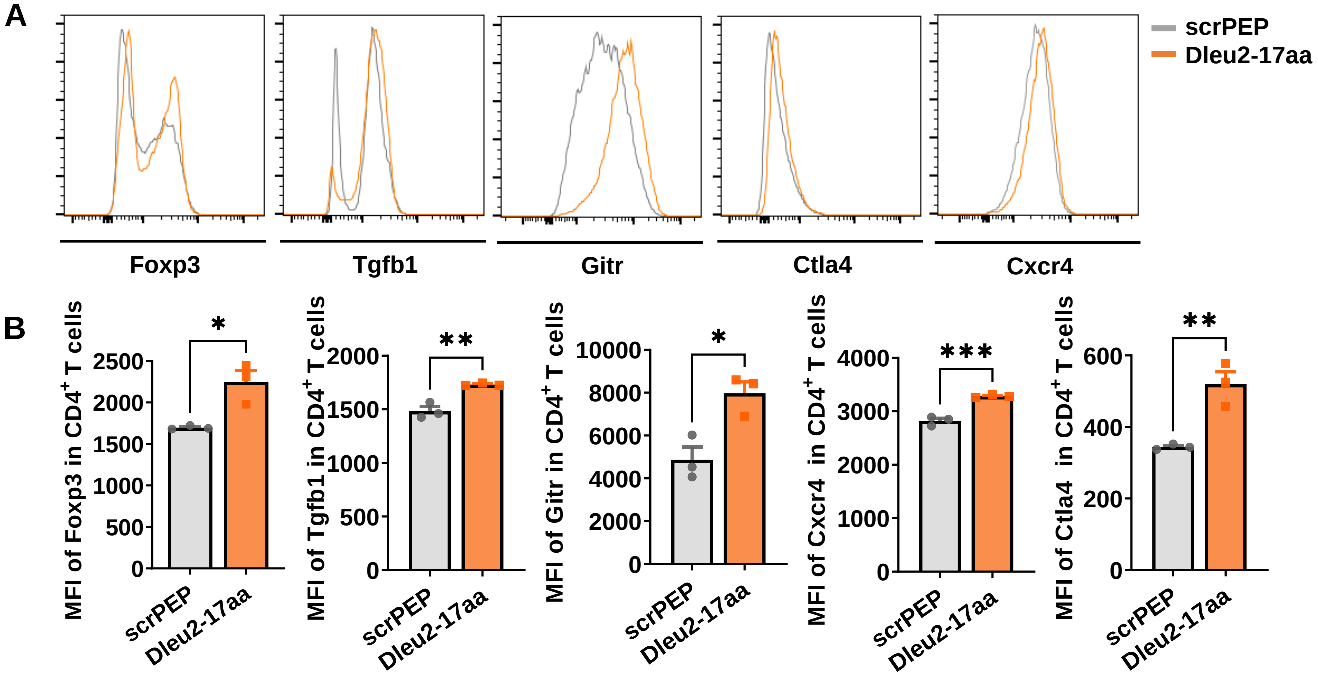

Appendix Fig S5: Dleu2-17aa enhances the expression of TGF-β/Smad signaling downstream genes and iTreg signature genes.

A. Representative histogram depicting the expression of TGF-β/Smad signaling downstream genes in $CD4^+$ T cells. The cells were challenged with TGF-β for 24 h to activate the signaling pathway and treated with Dleu2-17aa or scrPEP (n=3).

B. Median fluorescence intensity of proteins corresponding to the gene expression levels shown in (A) in $CD4^+$ T cells. The cells were challenged with TGF-β for 24 h to activate the signaling pathway and treated with Dleu2-17aa or scrPEP (n=3).

Data information: n indicates biological replicate. Error bars are mean ± SEM. *P<0.05, **P<0.01, and ***P < 0.001. Statistical analysis was by two-tailed Student's t-test for (B).

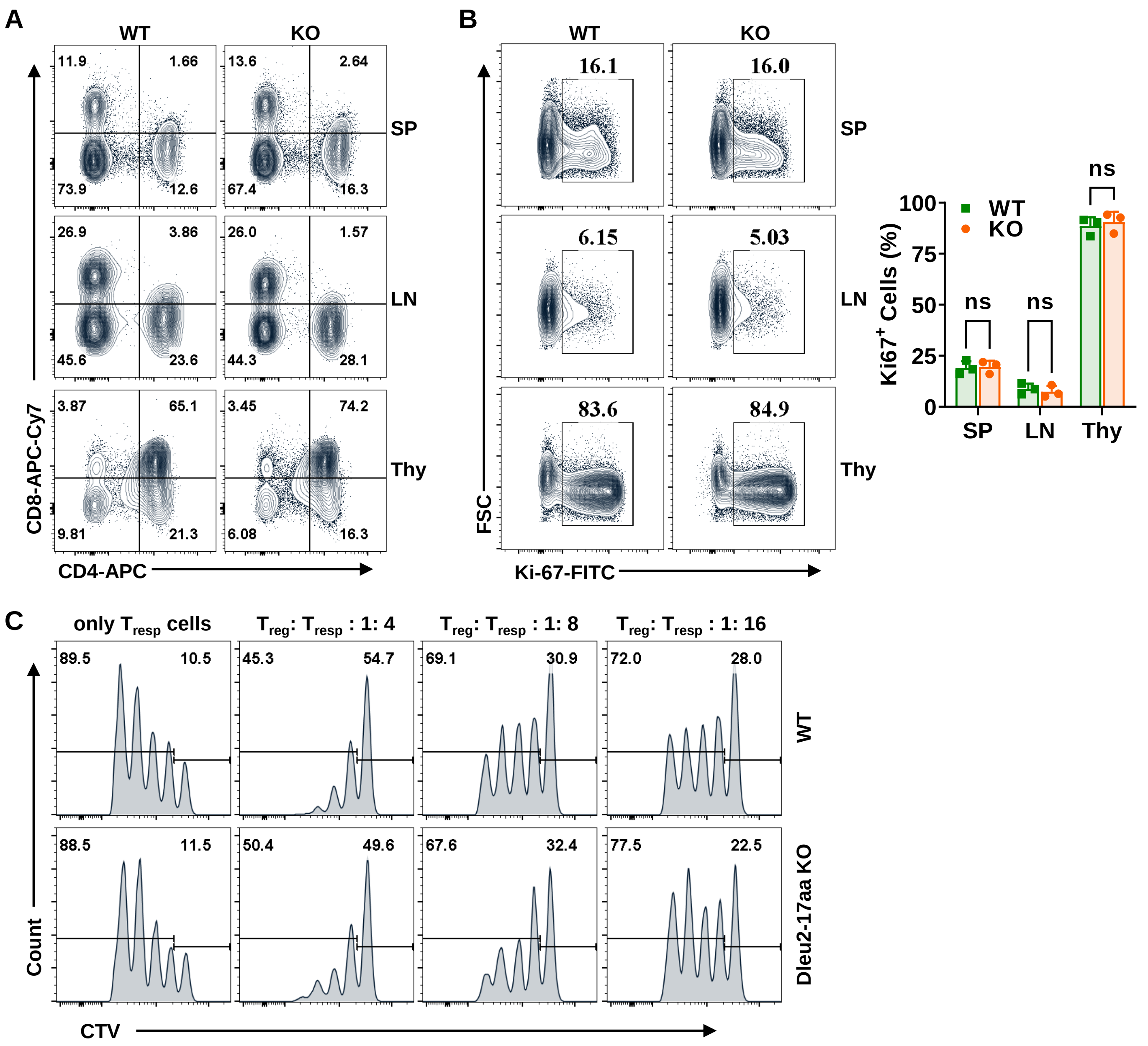

Appendix Fig S6: Effects of endogenous Dleu2-17aa on T cell development and function.

A. Representative flow cytometry depicting the expression of CD4 and CD8 in LN, SP, and Thy of WT and KO mice (n=3).

B. Phenotype (left panel) and relative frequencies (right panel) of the Ki-67 expression in LN, SP, and Thy of WT and KO mice (n=3).

C. Naive $CD4^+$ T cells were sorted from 6- to 8-week-old WT or KO mice, labeled with Cell Trace Violet, together with a decreasing ratio of sorted $CD25^+$ Treg cells from WT splenocytes, in the presence of anti-CD3 plus γ-irradiated antigen-presenting cells (1×10^5). The dilution of Cell Trace Violet was assessed by flow cytometry 72h post-culture (n=3).

Data information: n indicates biological replicate. Error bars are mean ± SEM. n.s. indicates no significant difference. Statistical analysis was by two-tailed Student's t-test for (B).

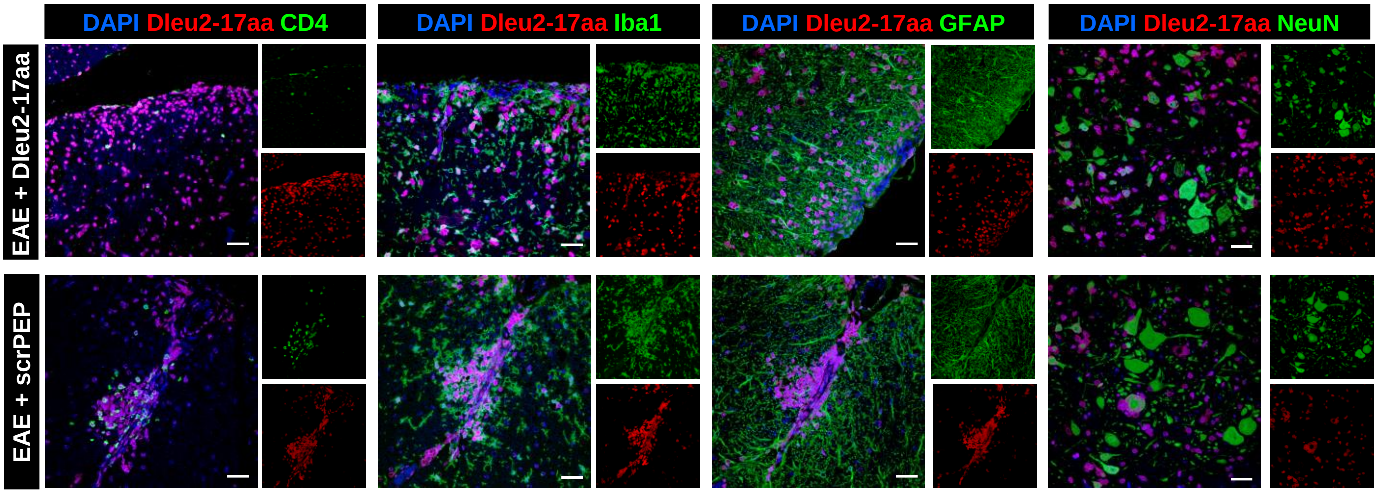

Appendix Fig S7: Effects of Dleu2-17aa on CNS pathology.

Representative immunofluorescence images showing Dleu2-17aa and $CD4^+$ T cells (CD4), microglia (Iba1), astrocyte (GFAP), and neuron (NeuN) staining in spinal cords of WT and KO EAE mice. Scale bar = 10 µm.

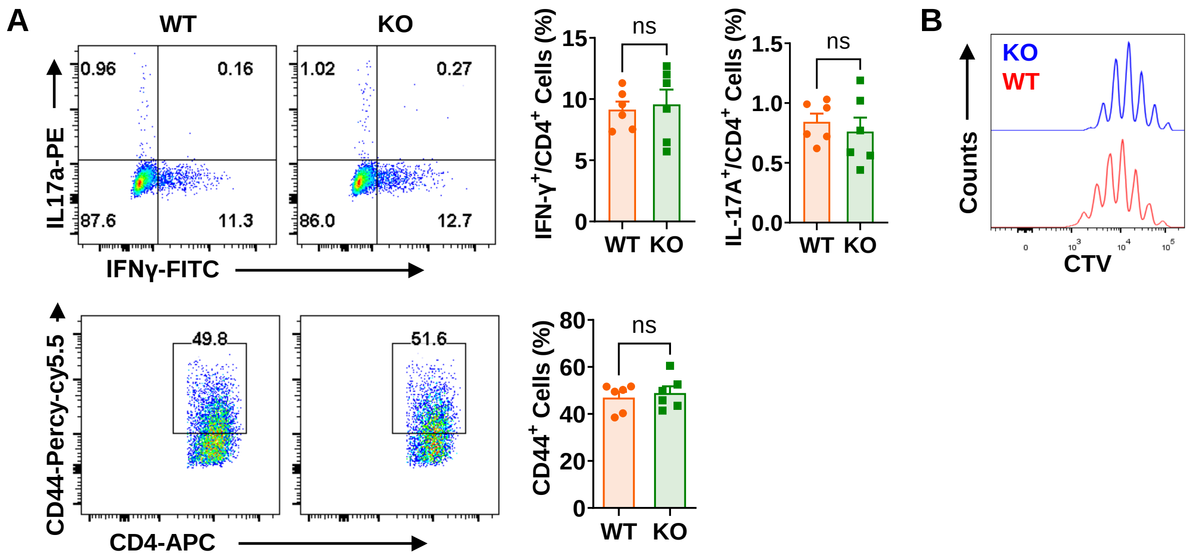

Appendix Fig S8: Effects of Dleu2-17aa on T cell recall response.

A. Phenotype (left panel) and relative frequencies (right panel) of the IFNγ and IL-17A expression in $CD4^{+}$ T cells from WT and KO EAE mice after *ex vivo* re-stimulation with $MOG_{35\text{-}55}$ peptide for 72 hours. (n=6-8).

B. Assessment of $CD4^{+}$ T cell proliferation by CTV dye dilution analysis after $MOG_{35\text{-}55}$ re-stimulation in WT and KO EAE mice. (n=3).

Data information: n indicates biological replicate. Error bars are mean ± SEM. n.s. indicates no significant difference. Statistical analysis was by two-tailed Student's t-test for (A).
