# Supplementary figures and images for: A lncRNA Dleu2-encoded peptide relieves autoimmunity by facilitating Smad3-mediated Treg induction
Source: EMBO Rep. 2024 Jan 30;25(3):18. doi: 10.1038/s44319-024-00070-4 (PMC10933344; doi:10.1038/s44319-024-00070-4)

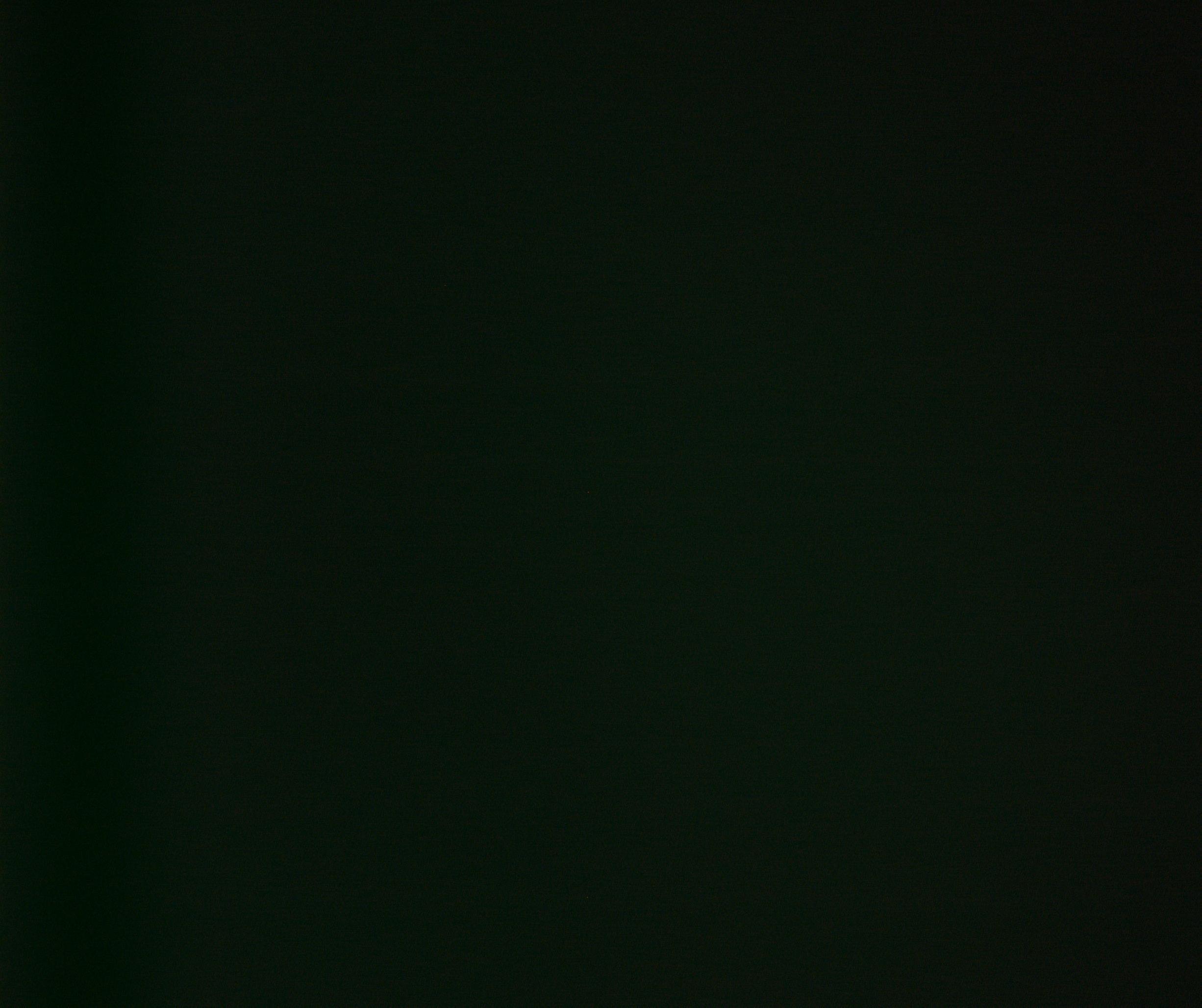

Supplement: Supplementary file 3 — Source Data Fig. 1 [file 44319_2024_70_MOESM3_ESM.zip › Figure 1/Figure 1E/MutORF-EGFP-ATT-200x.tiff]

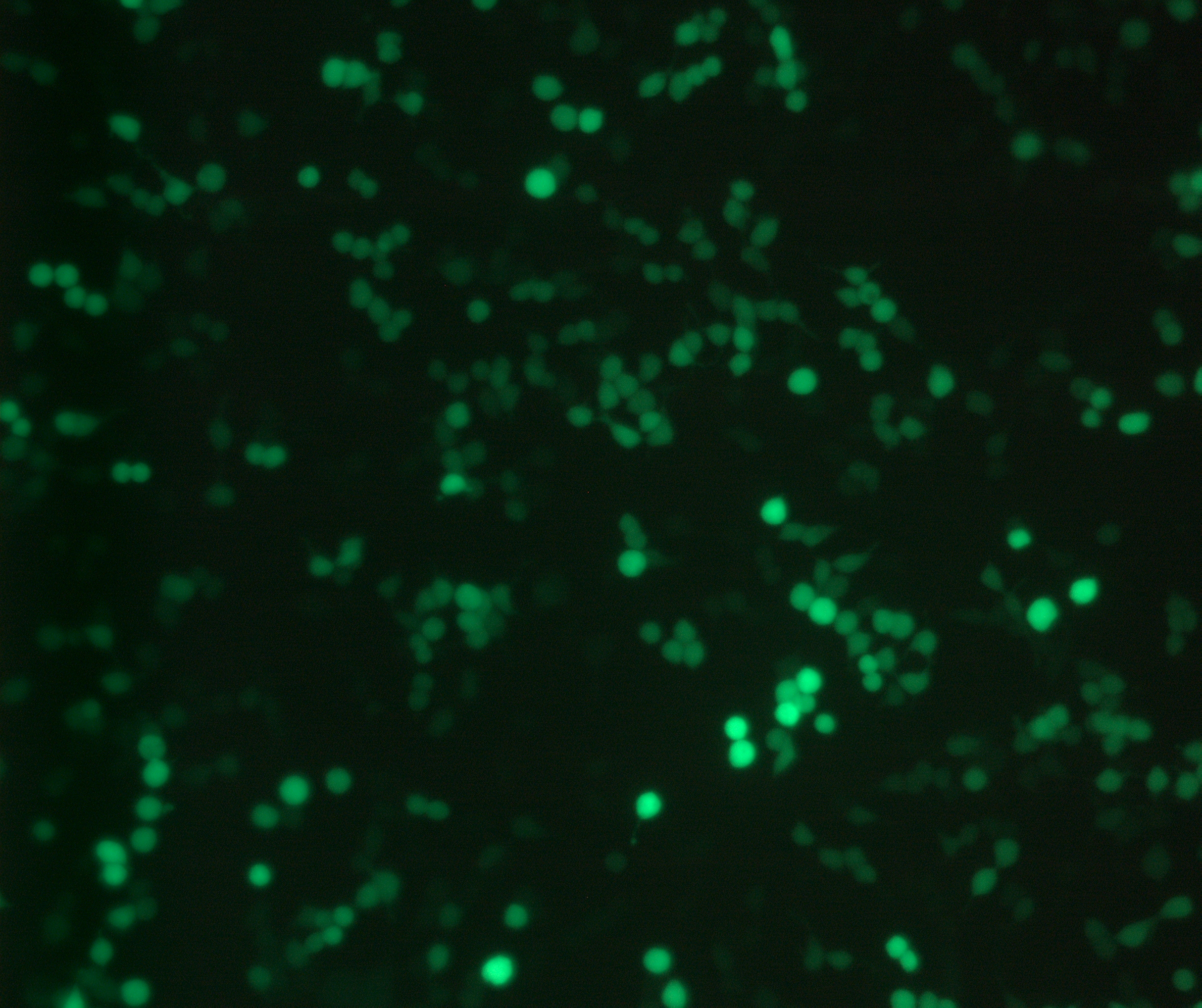

Supplement: Supplementary file 3 — Source Data Fig. 1 [file 44319_2024_70_MOESM3_ESM.zip › Figure 1/Figure 1E/ORF-EGFP-ATG-200x.tiff]

Figure 1 panel F

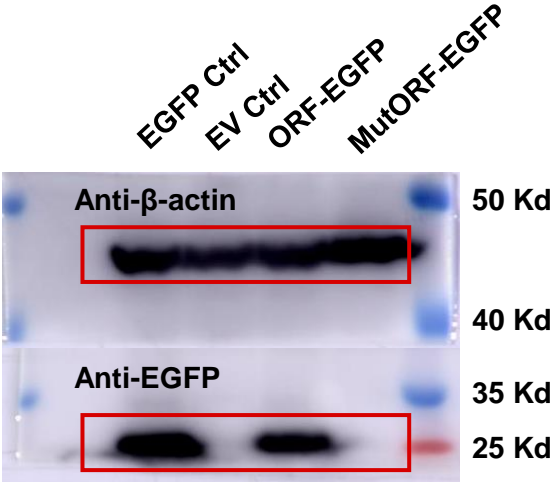

Figure 1 panel H

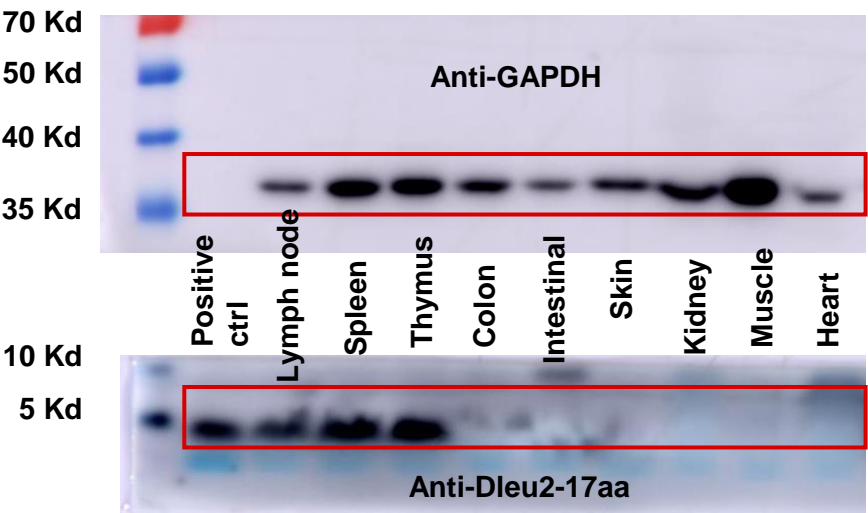

Supplement: Supplementary file 3 — Source Data Fig. 1 [file 44319_2024_70_MOESM3_ESM.zip › Figure 1/Figure 1F and H western blot.pdf]

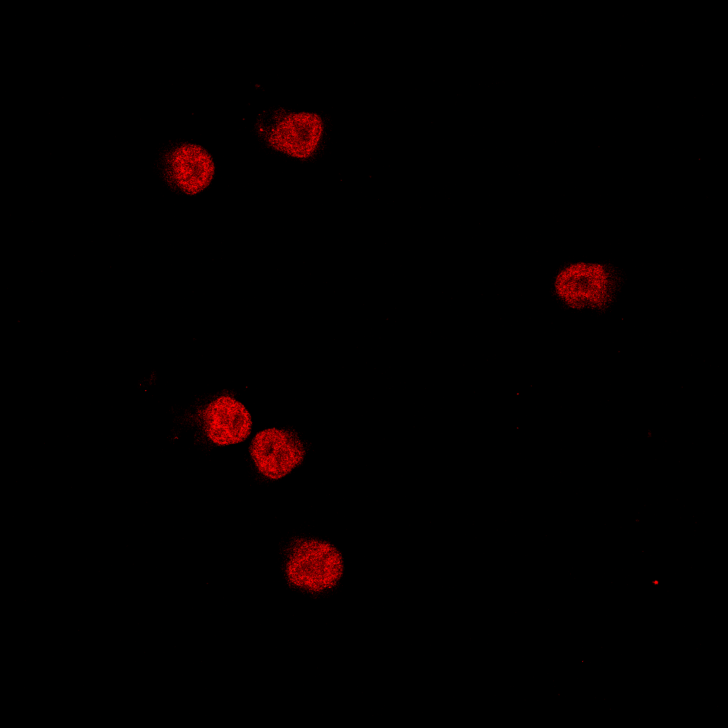

Supplement: Supplementary file 5 — Source Data Fig. 3 [file 44319_2024_70_MOESM5_ESM.zip › Figure 3/Figure 3A/Anti-17aa.tiff]

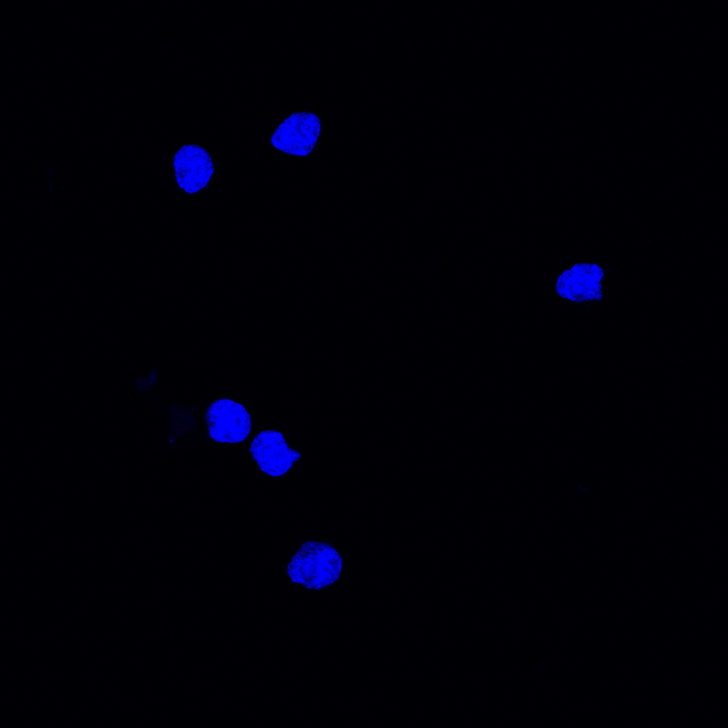

Supplement: Supplementary file 5 — Source Data Fig. 3 [file 44319_2024_70_MOESM5_ESM.zip › Figure 3/Figure 3A/DAPI.tiff]

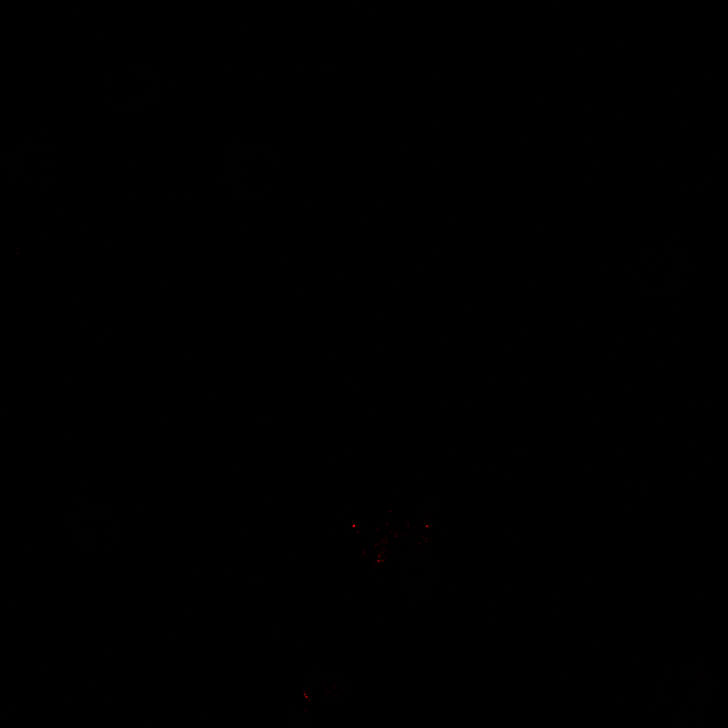

Supplement: Supplementary file 5 — Source Data Fig. 3 [file 44319_2024_70_MOESM5_ESM.zip › Figure 3/Figure 3A/isotype_CTRL Anti-17aa.tiff]

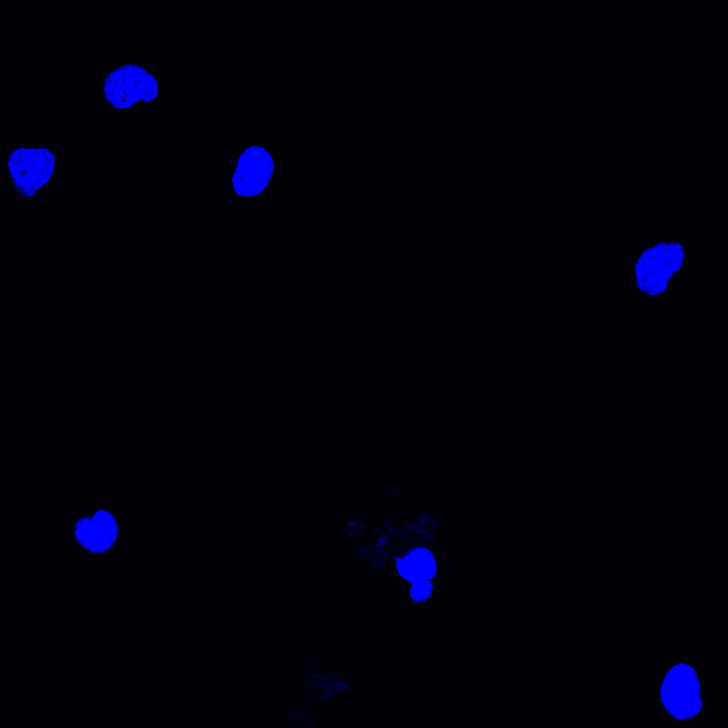

Supplement: Supplementary file 5 — Source Data Fig. 3 [file 44319_2024_70_MOESM5_ESM.zip › Figure 3/Figure 3A/isotype_CTRL DAPI.tiff]

Figure 3 panel D

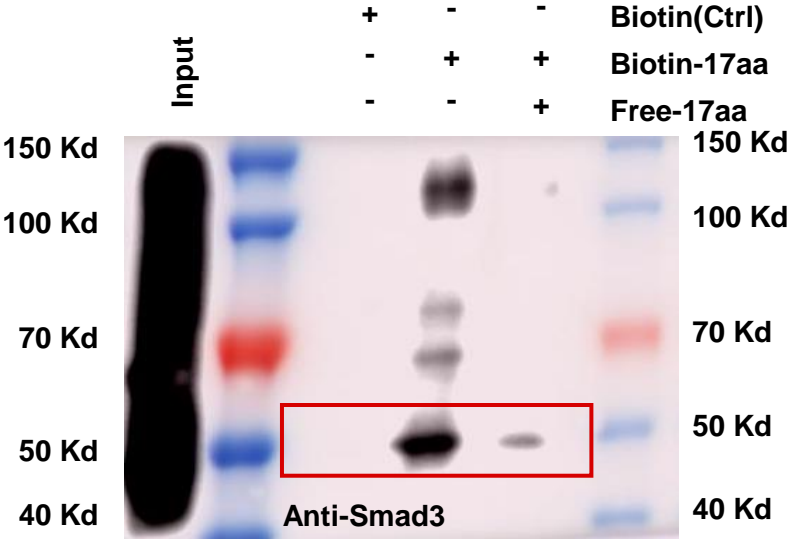

Supplement: Supplementary file 5 — Source Data Fig. 3 [file 44319_2024_70_MOESM5_ESM.zip › Figure 3/Figure 3D western blot.pdf]

Figure 4 panel D

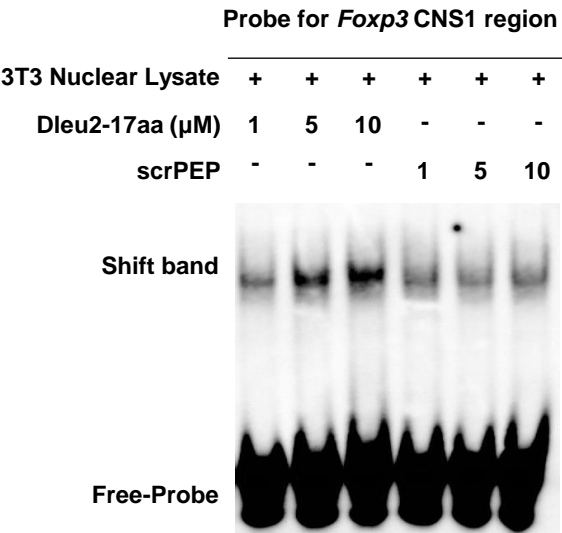

Supplement: Supplementary file 6 — Source Data Fig. 4 [file 44319_2024_70_MOESM6_ESM.zip › Figure 4/Figure 4D EMSA.pdf]

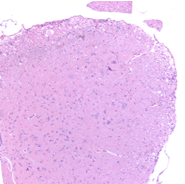

Supplement: Supplementary file 7 — Source Data Fig. 5 [file 44319_2024_70_MOESM7_ESM.zip › Source Data Figure 5/Figure 5C/Dleu2-17aa EAE HE.tif]

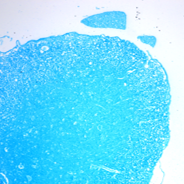

Supplement: Supplementary file 7 — Source Data Fig. 5 [file 44319_2024_70_MOESM7_ESM.zip › Source Data Figure 5/Figure 5C/Dleu2-17aa EAE LFB.tif]

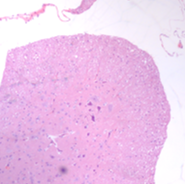

Supplement: Supplementary file 7 — Source Data Fig. 5 [file 44319_2024_70_MOESM7_ESM.zip › Source Data Figure 5/Figure 5C/Glatiramer EAE HE.tif]

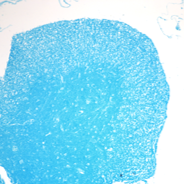

Supplement: Supplementary file 7 — Source Data Fig. 5 [file 44319_2024_70_MOESM7_ESM.zip › Source Data Figure 5/Figure 5C/Glatiramer EAE LFB.tif]

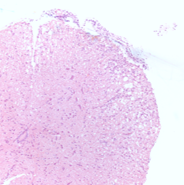

Supplement: Supplementary file 7 — Source Data Fig. 5 [file 44319_2024_70_MOESM7_ESM.zip › Source Data Figure 5/Figure 5C/scrPEP EAE HE.tif]

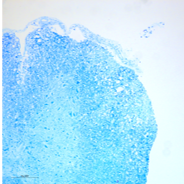

Supplement: Supplementary file 7 — Source Data Fig. 5 [file 44319_2024_70_MOESM7_ESM.zip › Source Data Figure 5/Figure 5C/scrPEP EAE LFB.tif]

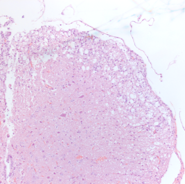

Supplement: Supplementary file 9 — Source Data Fig. 7 [file 44319_2024_70_MOESM9_ESM.zip › Source Data Figure 7/Figure 7C/KO EAE HE.tif]

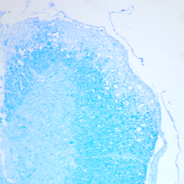

Supplement: Supplementary file 9 — Source Data Fig. 7 [file 44319_2024_70_MOESM9_ESM.zip › Source Data Figure 7/Figure 7C/KO EAE LFB.tif]

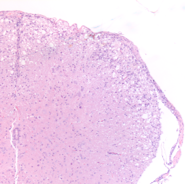

Supplement: Supplementary file 9 — Source Data Fig. 7 [file 44319_2024_70_MOESM9_ESM.zip › Source Data Figure 7/Figure 7C/WT EAE HE.tif]

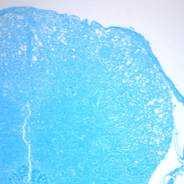

Supplement: Supplementary file 9 — Source Data Fig. 7 [file 44319_2024_70_MOESM9_ESM.zip › Source Data Figure 7/Figure 7C/WT EAE LFB.tif]
