# Supplementary material for: A lncRNA Dleu2-encoded peptide relieves autoimmunity by facilitating Smad3-mediated Treg induction
Source: EMBO Rep. 2024 Jan 30;25(3):18. doi: 10.1038/s44319-024-00070-4 (PMC10933344; doi:10.1038/s44319-024-00070-4)
Supplement: Supplementary file 11 — Expanded View Figures [file 44319_2024_70_MOESM11_ESM.pdf]

Expanded View Figures

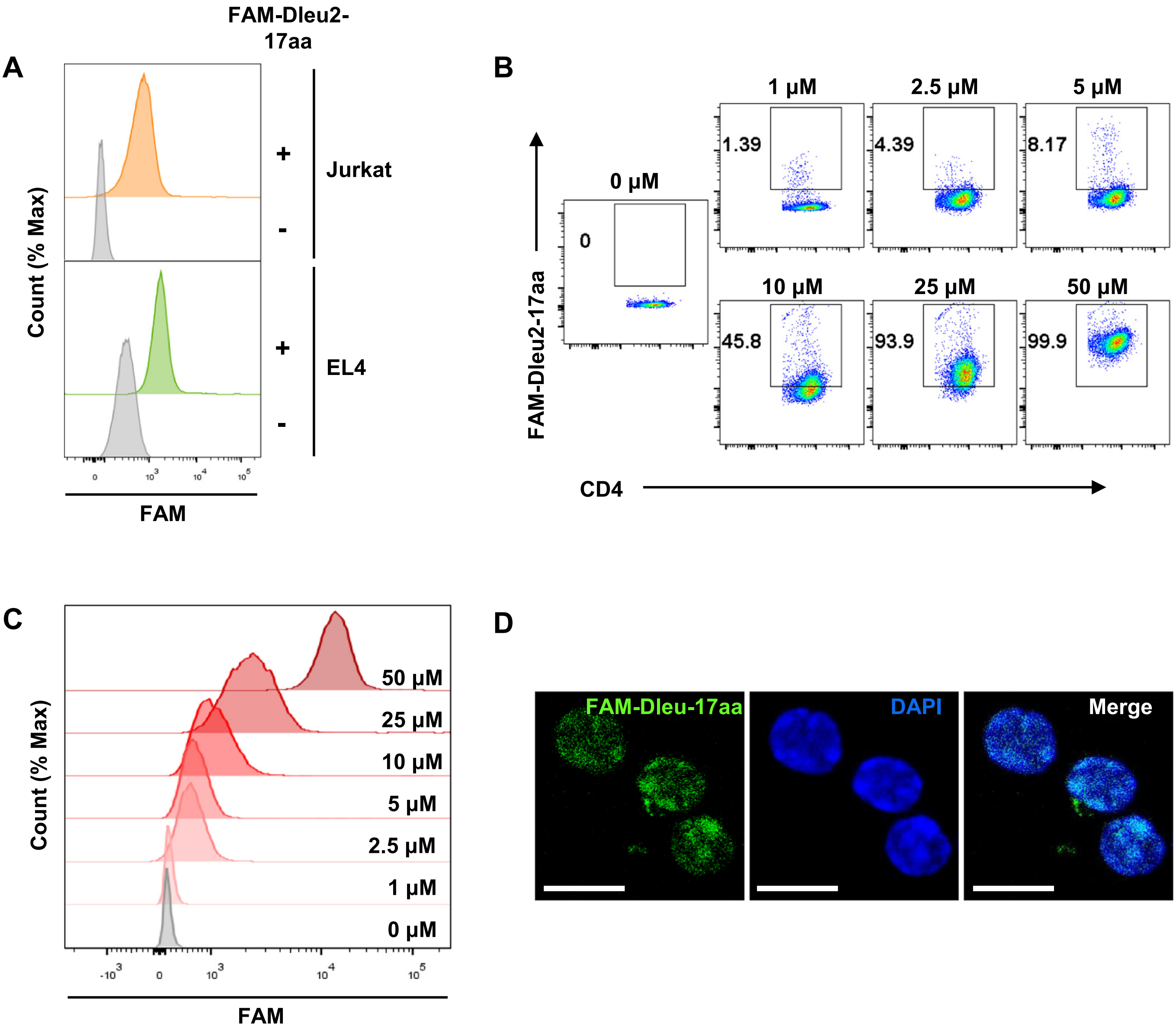

Figure EV1. Dleu2-17aa enters CD4+ T cells.

(**A**) Representative histogram of Dleu2-17aa (10 µM) uptake in Jurkat and EL4 cell lines (n = 3). (**B**) Flow cytometry plots showing FAM-Dleu2-17aa+ cells gated on CD4+ T cells after treatment with increasing concentrations of FAM-Dleu2-17aa for 72 h (n = 3). (**C**) Histograms showing fluorescence intensity of FAM-Dleu2-17aa in CD4+ T cells after treatment with increasing concentrations of FAM-Dleu2-17aa for 72 h. Gray filled: untreated control (n = 3). (**D**) Immunofluorescence imaging of FAM-Dleu2-17aa (10 µM) penetration by CD4+ T cells isolated from WT mouse spleen (n = 3). Scale bars: 20 µm. Data information: n indicates biological replicate.

© The Author(s)

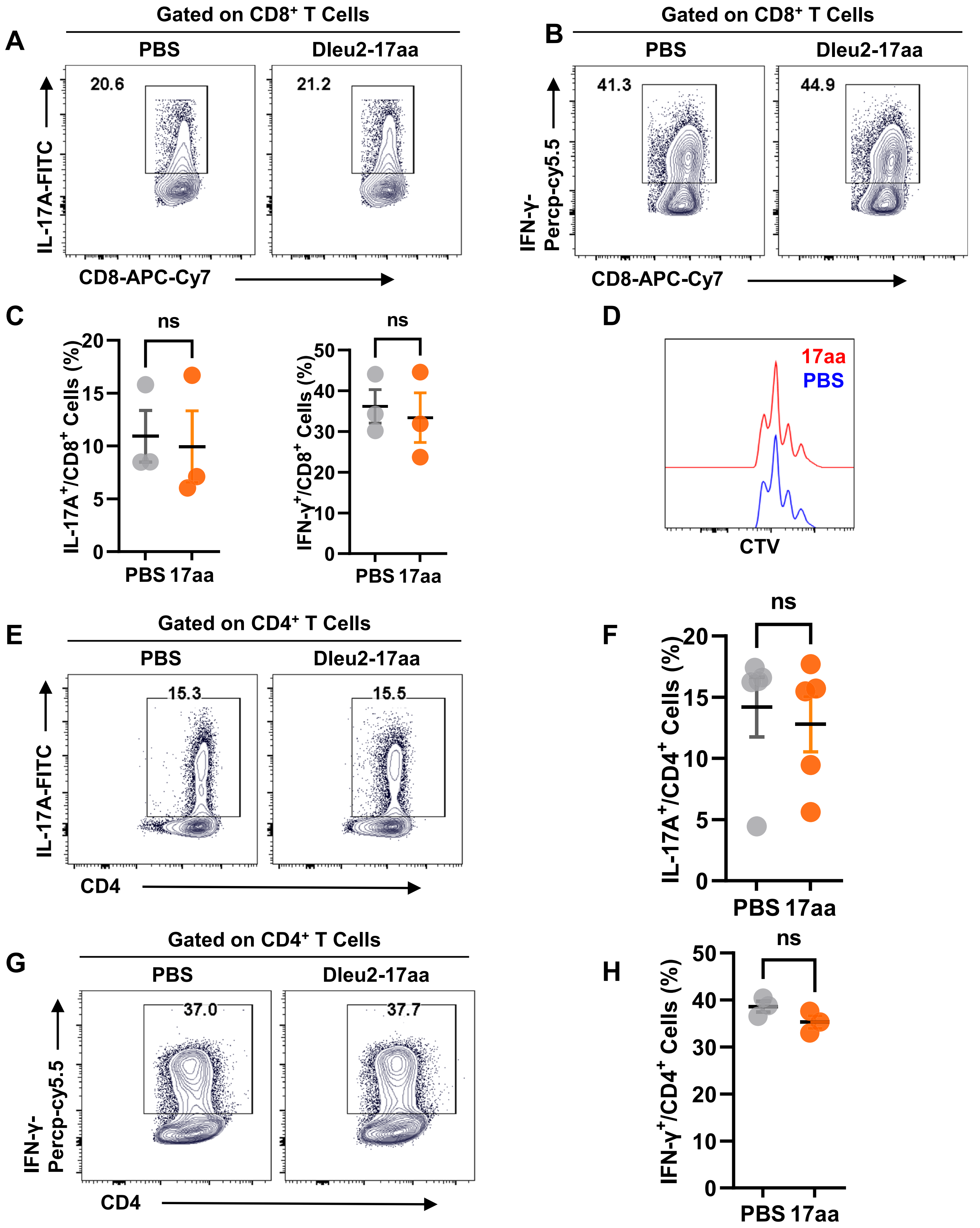
A
Gated on CD8⁺ T Cells
PBS
Dleu2-17aa
20.6
21.2
IL-17A-FITC
CD8-APC-Cy7
B
Gated on CD8⁺ T Cells
PBS
Dleu2-17aa
41.3
44.9
IFN-γ-Percp-cy5.5
CD8-APC-Cy7
C
ns
IL-17A⁺/CD8⁺ Cells (%)
PBS 17aa
ns
IFN-γ⁺/CD8⁺ Cells (%)
PBS 17aa
D
17aa
PBS
CTV
E
Gated on CD4⁺ T Cells
PBS
Dleu2-17aa
15.3
15.5
IL-17A-FITC
CD4
F
ns
IL-17A⁺/CD4⁺ Cells (%)
PBS 17aa
G
Gated on CD4⁺ T Cells
PBS
Dleu2-17aa
37.0
37.7
IFN-γ-Percp-cy5.5
CD4
H
ns
IFN-γ⁺/CD4⁺ Cells (%)
PBS 17aa

 © The Author(s)

Figure EV2. Effects of Dleu2-17aa on CD8$^+$ T cells and Th17 and Th1 cell differentiation.

(**A–C**) Phenotype (**A**,**B**) and relative frequencies (**C**) of IL-17A$^+$ Tc17 cells (**A**), and IFN-γ$^+$ Tc1 cells (**B**) subsets in PBS and Dleu2-17aa treated CD8$^+$ T cells of WT mice. Sorted naive CD8$^+$ T cells were incubated with PBS or Dleu2-17aa (10 μM) for 72 h under Tc1 or Tc17 induction conditions before analyzing cytokine expression in live cells ($n = 3$). (**D**) Representative flow cytometry of CTV histograms for CD8$^+$ T cells stimulated with anti-CD3 and anti-CD28 and cultured with either PBS or Dleu2-17aa (10 μM) for 72 h ($n = 3$). (**E–H**) Phenotype (left panel) and relative frequencies (right panel) of IL-17A$^+$ Th17 cells (**E**,**F**), IFN-γ$^+$ Th1 cells (**G**,**H**) subsets in PBS and Dleu2-17aa treated CD4$^+$ T cells of WT mice. Sorted naive CD4$^+$ T cells were incubated with PBS or Dleu2-17aa (10 μM) for 72 h under Th1 or Th17 induction conditions before analyzing cytokine expression in live cells ($n = 3$–5). Data information: n indicates biological replicate. Error bars are mean ± SEM; n.s. indicates no significant difference. Statistical analysis was by two-tailed Student's t-test for (**C**,**F**,**H**).

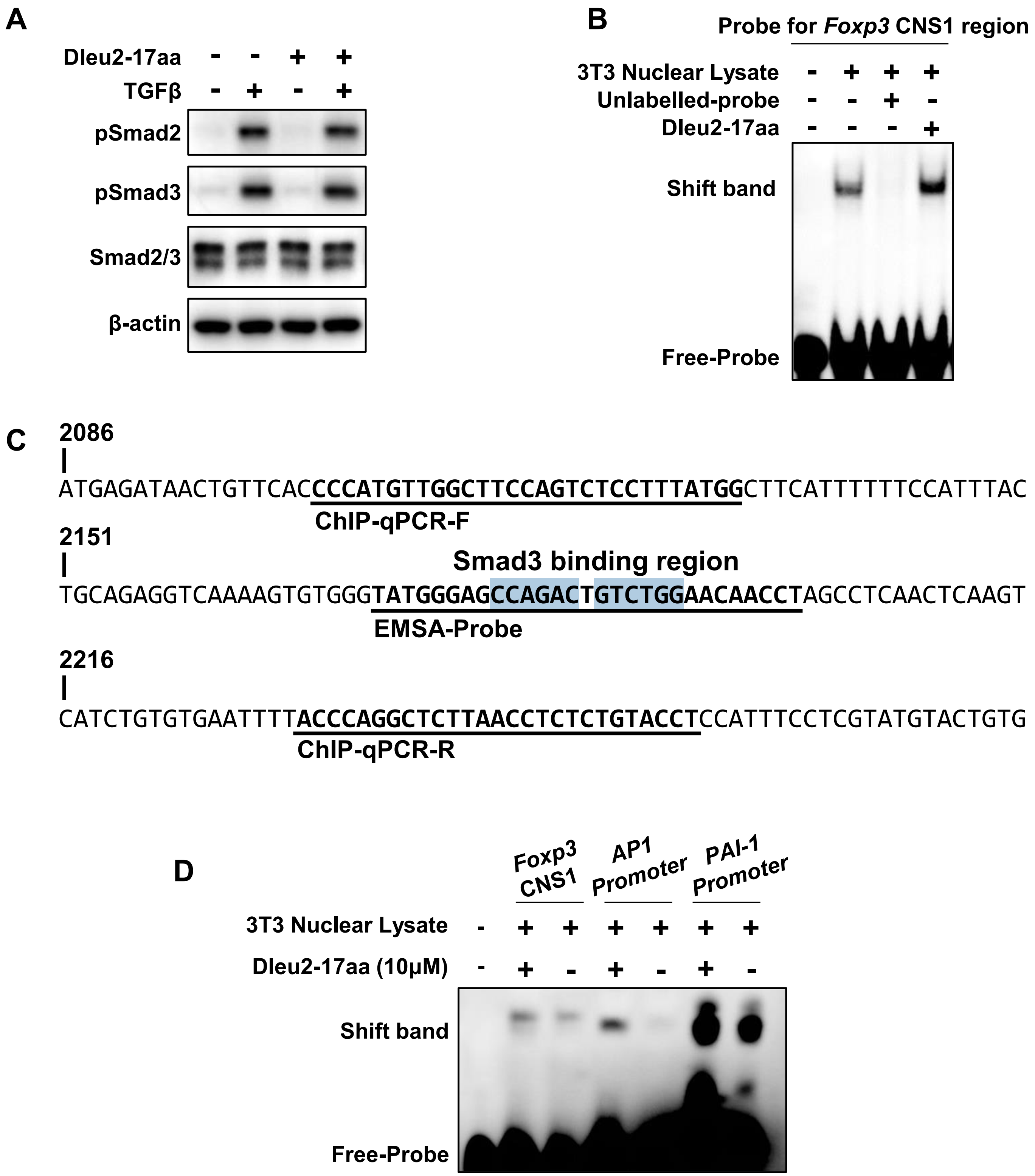
A
Dleu2-17aa - - + +
TGFβ - + - +
pSmad2
pSmad3
Smad2/3
β-actin
B
Probe for Foxp3 CNS1 region
3T3 Nuclear Lysate - + + +
Unlabelled-probe - - + -
Dleu2-17aa - - - +
Shift band
Free-Probe
C
2086
ATGAGATAACTGTTCACCCCATGTTGGCTTCCAGTCTCCTTTATGGCTTCATTTTTCCATTTAC
ChIP-qPCR-F
2151
Smad3 binding region
TGCAGAGGTCAAAAGTGTGGGTATGGGAGCCAGACTGTCTGGAACAACCTAGCCTCAACTCAAGT
EMSA-Probe
2216
CATCTGTGTGAATTTTACCCAGGCTCTTAACCTCTCTGTACCTCCATTTCCTCGTATGTACTGTG
ChIP-qPCR-R
D
Foxp3 CNS1
AP1 Promoter
PAI-1 Promoter
3T3 Nuclear Lysate - + + + + + +
Dleu2-17aa (10μM) - + - + - + -
Shift band
Free-Probe

 © The Author(s)

Figure EV3. Dleu2-17aa promotes Smad3 binding to the *Foxp3* CNS1 region.

(**A**) Western blot showing Total Smad2/Smad3 (lower blot) and phosphorylation of Smad2/Smad3 (upper blot) in $CD4^+$ T cells treated with TNF-β and/or Dleu2-17aa ($n = 3$). (**B**) EMSA was performed in NIH/3T3 cell nuclear lysates using a 10 μM biotin-labeled Foxp3 probe and treated with Dleu2-17aa (10 μM) or vehicle control. There were band shifts to a higher molecular weight and an increase in band intensity after Dleu2-17aa treatment ($n = 3$). (**C**) The CNS1 region of the *Foxp3* gene where Smad3 binds to. The EMSA probe and ChIP-qPCR primers used were pointed out in the figure. The Smad3 binding region was highlighted in blue. (**D**) EMSA was performed to assess the binding interactions between Dleu2-17aa and specific DNA sequences within NIH/3T3 cell nuclear lysates. 10 μM of different biotin-labeled probe were employed for the assay. Following treatment with Dleu2-17aa (10 μM) or vehicle control. There were band shifts to a higher molecular weight and an increase in band intensity after Dleu2-17aa treatment ($n = 3$). Data information: n indicates biological replicate.

© The Author(s)

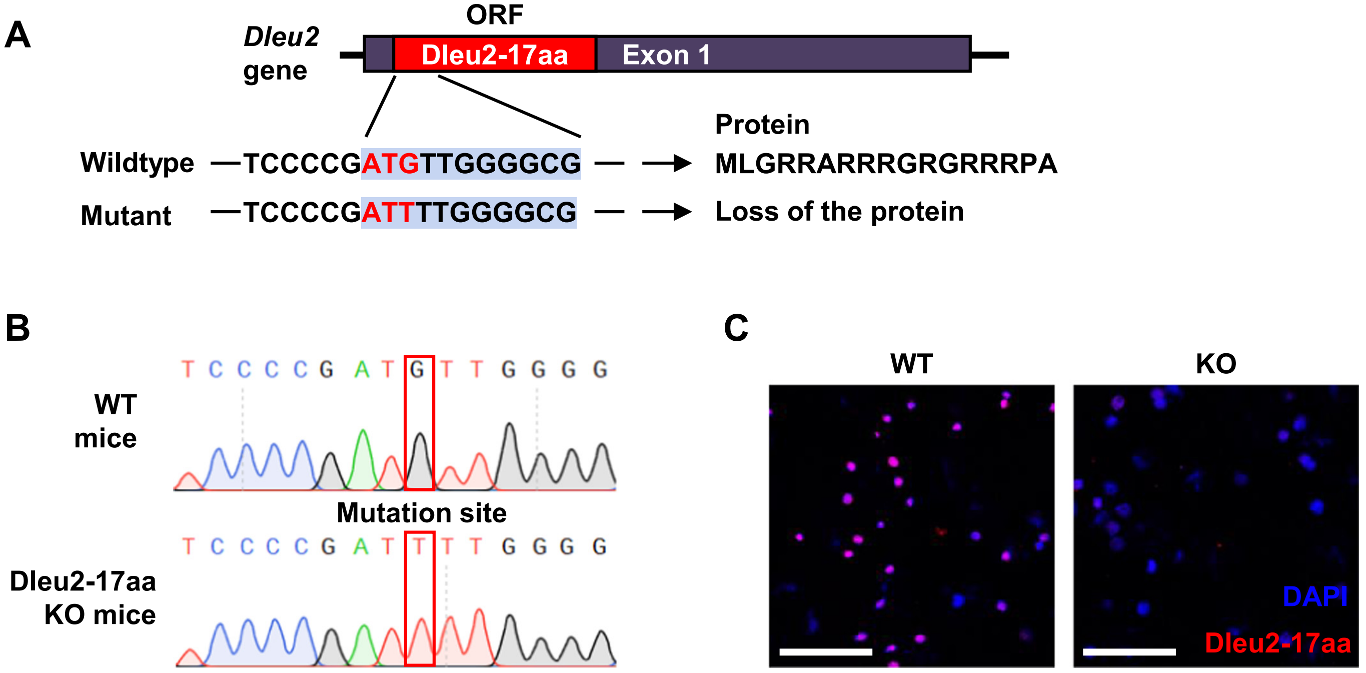

Figure EV4. Construction of Dleu2-17aa knockout mice.

(**A**) Schematic of the Dleu2-17aa mutant construct. The start codon ATG of Dleu2-17aa was mutated into ATT. (**B**) Sanger sequencing result of Dleu2-17aa locus from genomic DNA of the WT and KO mouse. (**C**) Representative immunofluorescence imaging of endogenous Dleu2-17aa expression in $CD4^{+}$ T cells of WT and KO mice ($n = 3$). Scale bars: 100 μm. Data information: n indicates biological replicate.

© The Author(s)

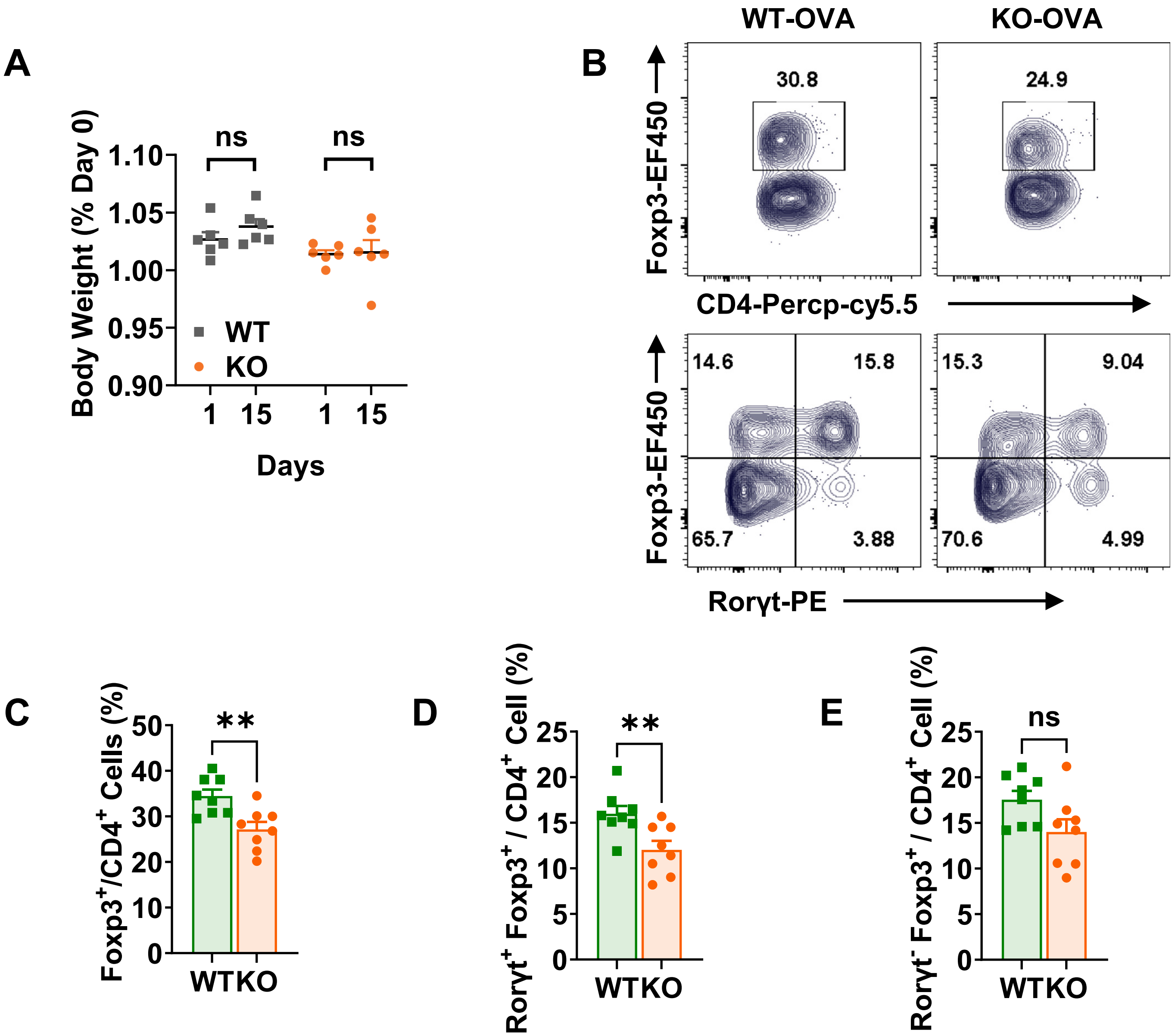

Figure EV5. Effects of Dleu2-17aa on dietary antigen induced pTreg differentiation.

(**A**) Body weight of day 1 and day 15 after oral gavage with OVA antigen and CTB ($n = 6$–8). (**B**) Representative flow plots showing Foxp3 and RORγt expression gated on CD4$^+$ cells from colon of WT and KO mice after 15 days OVA and CTB oral gavage ($n = 6$–8). (**C–E**) Summary graph depicting the percentage of Foxp3$^+$ cells (**C**), RORγt$^+$Foxp3$^+$ cells (**D**), RORγt$^-$Foxp3$^+$ cells (**E**) among total CD4$^+$ T cells in WT and KO mice cLN after 15 days OVA and CTB oral gavage ($n = 6$–8). Data information: n indicates biological replicate. Error bars are mean ± SEM; **$P < 0.01$, n.s. indicates no significant difference. Statistical analysis was by two-tailed Student's t-test for (**A**,**C**,**D**,**E**).

© The Author(s)
